# Differences in cancer survival by area-level socio-economic disadvantage: A population-based study using cancer registry data

**DOI:** 10.1371/journal.pone.0228551

**Published:** 2020-01-30

**Authors:** Nina Afshar, Dallas R. English, Tony Blakely, Vicky Thursfield, Helen Farrugia, Graham G. Giles, Roger L. Milne

**Affiliations:** 1 Cancer Epidemiology Division, Cancer Council Victoria, Melbourne, Victoria, Australia; 2 Centre for Epidemiology and Biostatistics, Melbourne School of Population and Global Health, The University of Melbourne, Melbourne, Victoria, Australia; 3 Victorian Cancer Registry, Cancer Council Victoria, Melbourne, Victoria, Australia; 4 Precision Medicine, School of Clinical Sciences at Monash Health, Monash University, Clayton, Victoria, Australia; Chang Gung Memorial Hospital at Linkou, TAIWAN

## Abstract

Despite overall improvements in cancer survival due to earlier diagnosis and better treatment, socio-economically disadvantaged people have lower cancer survival than more advantaged people. We aimed to examine differences in cancer survival by area-level socio-economic disadvantage in Victoria, Australia and assess whether these inequalities varied by year of diagnosis, age at diagnosis, time since diagnosis and sex. Cases diagnosed with a first primary cancer in 2001–2015 were identified using the Victorian Cancer Registry and followed to the end of 2016. Five-year net survival and the excess risk of death due to a cancer diagnosis were estimated. People living in more disadvantaged areas had lower five-year survival than residents of less disadvantaged regions for 21 of 29 cancer types: head and neck, oesophagus, stomach, colorectum, anus/anal canal, liver, gallbladder/biliary tract, pancreas, lung, melanoma, connective/soft tissue, female breast, ovary, prostate, kidney, bladder, brain and central nervous system, unknown primary, non-Hodgkin lymphoma, multiple myeloma and leukemia. The observed lower survival in more deprived regions persisted over time, except head and neck cancer, for which the gap in survival has widened. Socio-economic inequalities in survival decreased with increasing age at diagnosis for cancers of connective/soft tissue, bladder and unknown primary. For colorectal cancer, the observed survival disadvantage in lower socio-economic regions was greater for men than for women, while for brain and central nervous system tumours, it was larger for women. Cancer survival is generally lower for residents of more socio-economically disadvantaged areas. Identifying the underlying reasons for these inequalities is important and may help to identify effective interventions to increase survival for underprivileged cancer patients.

## Introduction

Over recent decades, survival has increased for most cancer types due to earlier diagnosis and more effective treatments. [1, 2] In parallel, there is well-documented evidence that socio-economic inequalities in cancer survival exist in high-income countries, with disadvantaged cancer patients having lower survival than their counterparts with higher socio-economic position (SEP). [3–5] Even though Australia has a universal health care system, socio-economic differences in cancer survival exist. [6–8] In 2010–2014, five-year relative survival for all cancers combined was lower for patients living in the most disadvantaged areas (55%), relative to the least disadvantaged areas (67%). [9] The largest gaps were found for cancers of the head and neck, colorectum, cervix, kidney, prostate as well as non-Hodgkin lymphoma. [9]

Australian studies that have investigated socio-economic differences in cancer survival have not used socio-economic status-specific life tables to estimate relative survival [8, 10] and may have provided biased survival estimates by overestimating relative survival gaps, as the expected background mortality of more disadvantaged people is likely to be underestimated in general population life tables (i.e. more deaths than should have been were attributed to cancer).

Several studies, including one from Australia, have observed differences in cancer survival by sex; men generally have lower survival than women for most cancers, [11–15] but it is not clear whether socio-economic inequalities in cancer survival differ between men and women. Additionally, published studies have not examined socio-economic inequalities by age at diagnosis or time since cancer diagnosis. We aimed to assess differences in cancer survival by area-level socio-economic disadvantage using the Victorian Cancer Registry data. We investigated whether these inequalities are widening or narrowing over time and examined differences by age at diagnosis, time since diagnosis and sex.

## Materials and methods

### Data sources

Our analyses of deidentified Victorian Cancer Registry (VCR) data was approved by the Cancer Council Victoria Human Research Ethics Committee.

Cases were identified from records of invasive cancers held by the VCR, a population-based registry in the state of Victoria, Australia which receives notifications from hospitals, pathology laboratories and cancer screening registries. Data items collected for each cancer include date of diagnosis, tumour anatomical location, morphology, grade and behaviour, as well as patient name, address, date of birth and sex. Information on vital status is routinely updated via linkage to the Victorian Registry of Births, Deaths and Marriages and the National Death Index at the Australian Institute of Health and Welfare.

The address of usual residence registered at the time of diagnosis was mapped and coded to the smallest geographical areas defined by the Australian Bureau of Statistics (ABS). For cancer cases diagnosed in 2009–2015 this was Statistical Area Level 1 (SA1; mean population size of 400 persons), [16] and for cases diagnosed in 2001–2008, this was Collection District (CD; mean of 225 dwellings). [17] An area-based measure of socio-economic disadvantage (Socio-Economic Indexes for Areas, SEIFA) was determined using the census-based Index of Relative Socio-economic Disadvantage (IRSD). [18] A low score on the IRSD indicates a high proportion of economically and socially disadvantaged people in an area (e.g. many households with low income, many people with no qualifications or in low-skill occupations). [18] The distribution of SEIFA index values across geographical areas in the Victorian population was used to define quintiles, and the order reversed so that quintile 1 included the least disadvantaged and quintile 5 the most disadvantaged.

The ABS constructed SEIFA-specific life tables by year, sex and single year of age for the period 2001–2015. Victorian population mortality rates at ABS Statistical Area Level 2 (SA2) were aggregated into quintiles. The mortality curves for all SEIFA quintiles were smoothed using the Hodrick-Prescott filter to reduce the variability of *q*_*x*_ values (i.e. the probability of dying at age x). [19]

### Study cohort

Patients were eligible if they were diagnosed with a first primary cancer of one of 29 types in the period from 1 January 2001 until 30 December 2015 and aged 15–99 at diagnosis (n = 331,419). We chose the 29 cancers for which at least 1,000 cases were reported to the VCR during the study period. Second primary neoplasms, in situ cancers, keratinocyte carcinomas and male breast cancers were not considered. We excluded patients notified only via autopsy or death certificate as well as those with missing data for area-level socio-economic disadvantage.

### Statistical analysis

We used the Pohar-Perme method with SEIFA-specific Victorian population life tables to calculate five-year net survival and 95% confidence intervals (CIs) for each quintile of socio-economic disadvantage. [20, 21] Net survival was estimated by first calculating the ratio of each patient’s observed survival relative to that expected for a member of the general population without cancer and of the same age, sex, calendar year and SEIFA quintile, and then averaging for all patients. [20] Expected survival was calculated using the Ederer II method. [22] To allow comparisons across socio-economic groups with differing distributions of age at diagnosis, we calculated age-standardised net survival using age-specific weights. For each cancer site, the weights were calculated based on the distribution of age at diagnosis of all cancers of that site using five age groups (15–44, 45–6, 65–74, and 75–99 years). Follow-up commenced at date of diagnosis and concluded five years later, on the date of death, or December 31, 2015, whichever came first. To avoid bias in the estimation of survival, one day of survival was given to cases with the same date of diagnosis and death. [23]

We modelled excess mortality rates due to cancer. [24] The excess mortality rate is the difference between the mortality rate for patients and the expected mortality rate for the general population. First, we calculated observed and expected rates by calendar year, age, sex, and SEIFA category and collapsed the resulting data by period of diagnosis (2001–2005, 2006–2010, 2011–2015), age at diagnosis (15–44, 45–64, 65–74, and 75–99 years), time since diagnosis (1st, 2nd, 3rd, 4th and 5th year) and sex. We then modelled these data using Poisson regression with each covariate included in the model, together with interaction terms between age group and time since diagnosis, as elderly cancer patients have higher excess risk of death within two years following diagnosis. [25] The exponentiated parameter estimates from these models are excess mortality rate ratios (EMRR). To interpret the EMRR, consider an EMRR of 1.4 comparing the most disadvantaged patients with the least disadvantaged. This value indicates that the excess mortality rate due to the cancer is 1.4 times higher for the most disadvantaged than for the least disadvantaged.

To assess heterogeneity in the EMRR, four additional models were run, fitting interactions between pseudo-continuous SEIFA (the quintiles were treated as continuous with 5 integer values) and each of (i) year of diagnosis, (ii) age at diagnosis, (iii) time since diagnosis, and (iv) sex. We conducted these analyses for cancer types that showed consistent trends in socio-economic differences in survival. We also stratified on these covariates.

People residing outside major cities are generally more disadvantaged and have been reported to have lower cancer survival. [9] Because the measures of remoteness and socio-economic disadvantage available were both area-based (using the same geographical areas), rather than adjusting for remoteness, we conducted a sensitivity analysis restricted to cases living in major cities as an attempt to remove any potentially confounding effects of factors associated with remoteness such as access to services.

We used Pearson’s goodness-of-fit statistic to compare models with different number of degrees of freedom. [26] A likelihood ratio test and a two-sided *p* value were applied to assess statistical significance. Stata/MP version 14.2 (Stata Corporation LP, College Station, TX, USA) was used to perform all statistical analyses.

## Results

**Fig 1:**
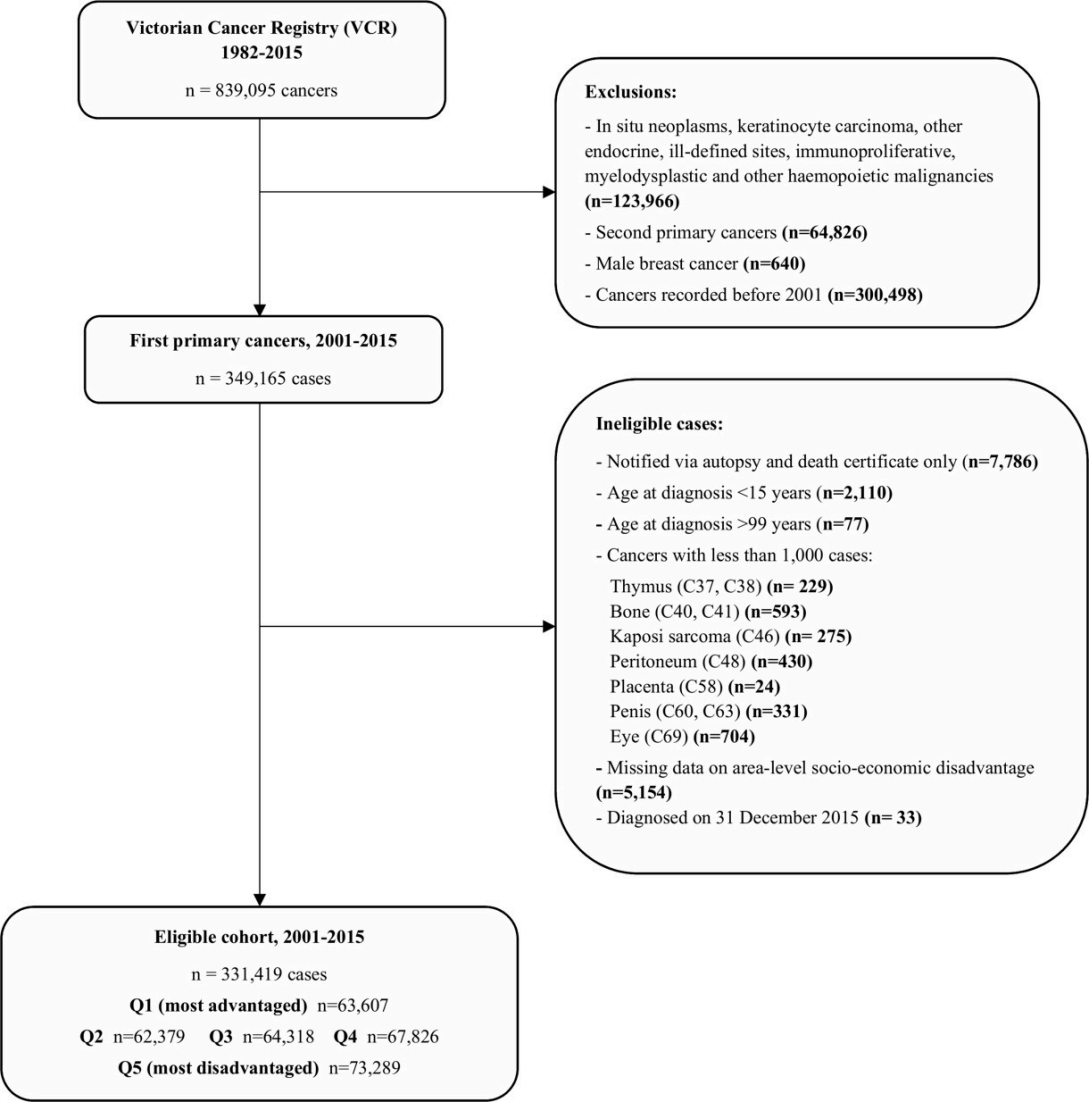
Case selection from the Victorian Cancer Registry.

A total of 331,419 Victorian residents diagnosed between January 1, 2001 and December 30, 2015 with one of the 29 incident cancers considered were included in the analyses (Fig 1). The number of cases and deaths for each cancer type, by area-level socio-economic disadvantage, are listed in S1 Table. The mean age at diagnosis was 63.5 years for patients living in the least disadvantaged areas and 66.7 years for cases from the most disadvantaged regions.

**Fig 2:**
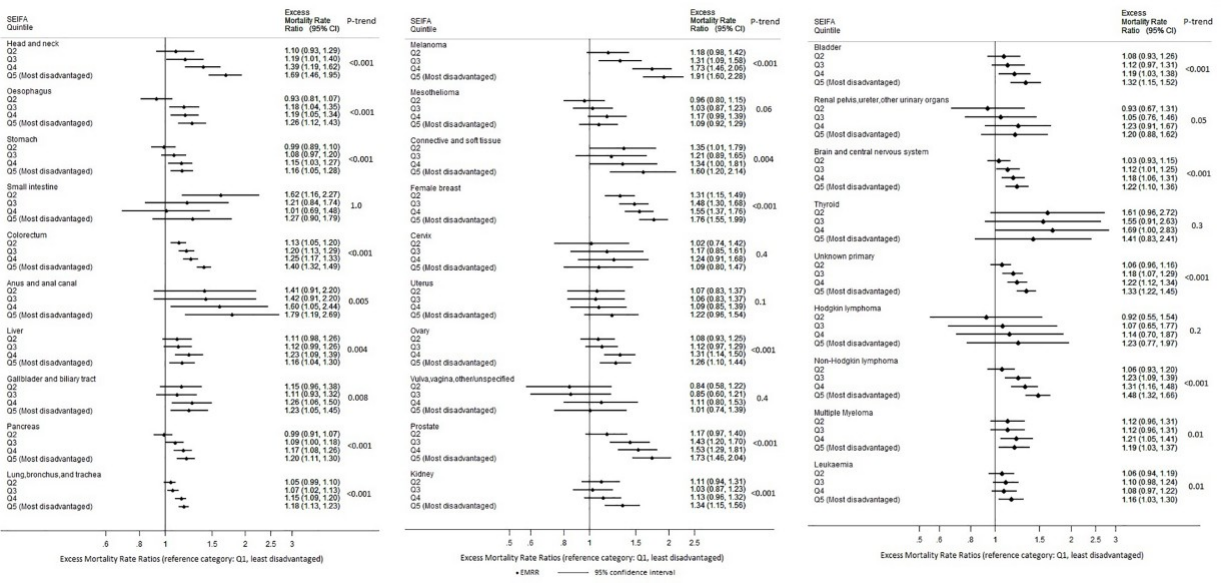
Excess Mortality Rate Ratios (EMRRs) within 5-year of diagnosis, by area-level socio-economic disadvantage, in Victoria, Australia, 2001–2015. Panel A: Cancer sites with ICD-10 C00-C34; Panel B: Cancer sites with ICD-10 C43-C63; Panel C: Cancer sites with ICD-10 C65-C96.

The EMRR and age-standardised five-year net survival estimates are presented in Fig 2 and S2 Table, respectively. For 21 of the 29 cancer types, a consistent trend of lower survival was observed for people living in more disadvantaged areas (*p*-trend <0.05 in Fig 2). The magnitude of these differences varied by cancer type (Fig 2), but was notable for the five most common cancers except lung cancer, with EMRR comparing the most with the least disadvantaged areas 1.40 (95%CI 1.32–1.49), 1.91 (95%CI 1.60–2.28), 1.76 (95%CI 1.55–1.99), 1.73 (95%CI 1.46–2.04) for colorectal cancer, melanoma, female breast and prostate cancer, respectively. The EMRR for the most relative to the least disadvantaged areas was also high for cancers of the head and neck (1.69; 95%CI 1.46–1.95), connective and soft tissue (1.60; 95% CI 1.20–2.14), kidney (1.34; 95%CI 1.15–1.56), bladder (1.32; 95%CI 1.15–1.52), unknown primary (1.33; 95%CI 1.22–1.45) and non-Hodgkin lymphoma (1.48; 95%CI 1.32–1.66). A similar pattern of lower survival in more disadvantaged areas was found for cancers of the oesophagus, stomach, anus/anal canal, liver, gallbladder/biliary tract, pancreas, lung, ovary, brain and central nervous system (CNS), multiple myeloma and leukaemia. For cancers of small intestine, mesothelioma, cervix, uterus, renal pelvis/ureter, thyroid and Hodgkin-lymphoma, there was some evidence of greater excess mortality in the most disadvantaged areas, but no clear trends across socio-economic groups (Fig 2). We observed no socio-economic differences in survival from cancer of vulva and vagina.

**Fig 3:**
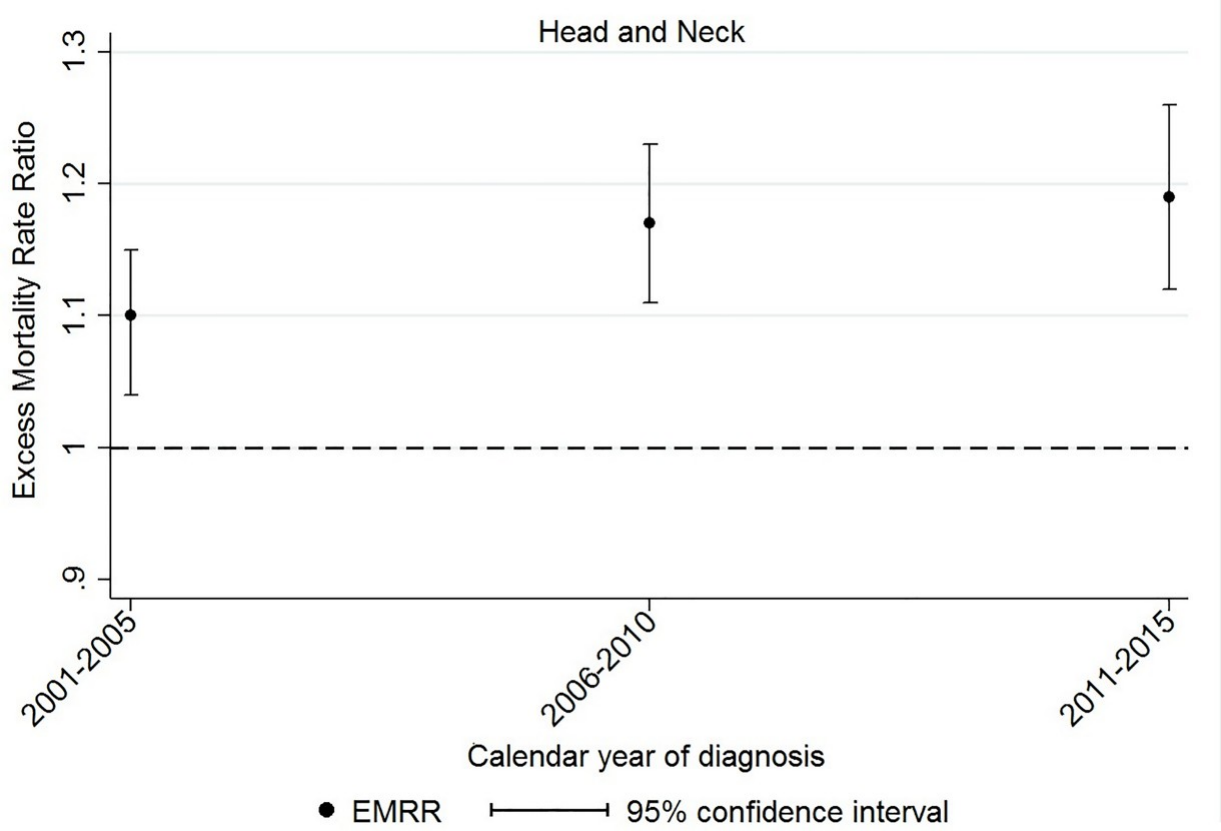
Five-year excess mortality rate ratios (EMRRs), by year of diagnosis, per quintile increase in socio-economic disadvantage (SEIFA). The dashed line shows the reference value for the EMRR, representing no differences in survival between the least disadvantaged areas and more disadvantaged regions.

There was no strong evidence of widening or narrowing gaps in survival over the study period except for head and neck cancer, for which differences in survival increased over time, from an EMRR per quintile increase in SEIFA-based disadvantage of 1.10 (95%CI 1.04–1.15) in 2001–2005 to 1.19 (95%CI 1.12–1.26) in 2011–2015 (S3 Table, Fig 3). Weak evidence of a similar pattern was noted for tumours of the brain and CNS. We also found weak evidence of decreasing differences in survival over time for non-Hodgkin lymphoma and oesophageal cancer (S3 Table).

**Fig 4:**
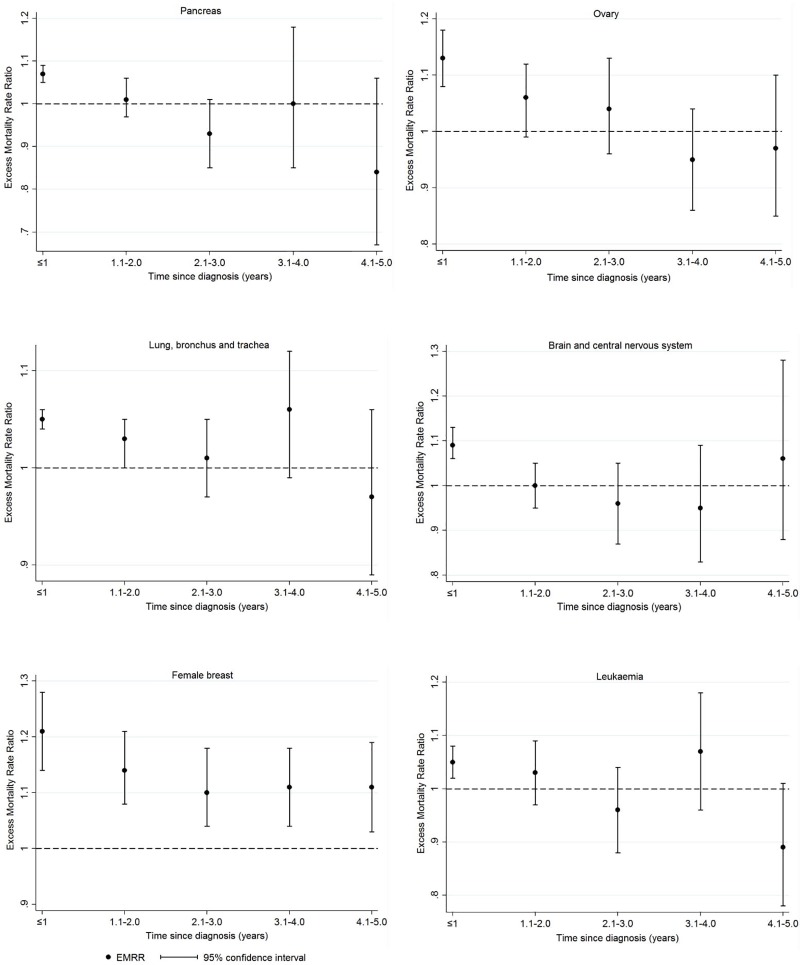
Five-year excess mortality rate ratios (EMRRs), by time since diagnosis, per quintile increase in socio-economic disadvantage (SEIFA). The dashed line shows the reference value for the EMRR, representing no differences in survival between the least disadvantaged areas and more disadvantaged regions.

For leukaemia and cancers of the pancreas and brain/CNS, lower survival for people living in more disadvantaged areas was mainly evident in the first year of diagnosis, while for lung and ovarian cancer, it was apparent within three years after diagnosis (S4 Table, Fig 4). For female breast cancer, socio-economic inequalities in survival decreased with time since diagnosis, but persisted over five years.

**Fig 5:**
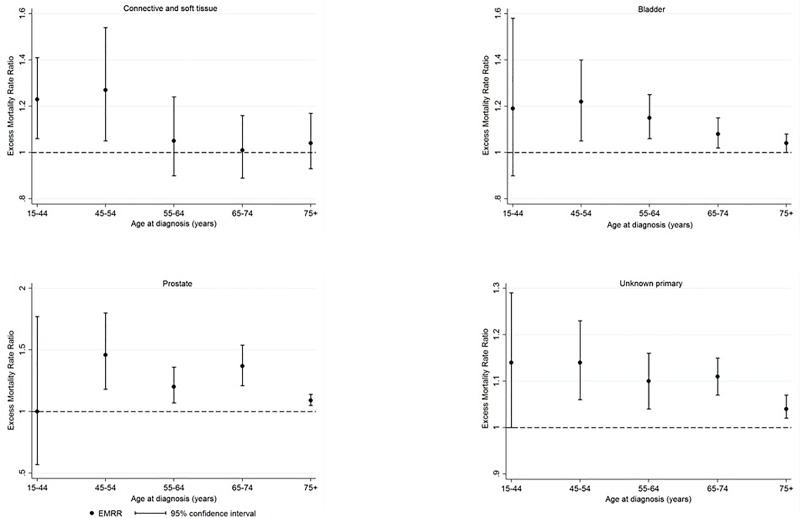
Five-year excess mortality rate ratios (EMRRs), by age at diagnosis, per quintile increase in socio-economic disadvantage (SEIFA). The dashed line shows the reference value for the EMRR, representing no differences in survival between the least disadvantaged areas and more disadvantaged regions.

For unknown primary cancer and bladder cancer, the relative excess mortality for cases from more disadvantaged areas became smaller with increasing age at diagnosis (S5 Table, Fig 5). For prostate cancer, excess mortality varied inconsistently by age. For connective and soft tissue cancer, differences in survival were only observed for younger patients (<55 years).

Socio-economic differences in survival after colorectal cancer were larger for men (EMRR per quintile increase in SEIFA 1.10; 95%CI 1.08–1.12) than for women (EMRR 1.06; 95%CI 1.04–1.08). In contrast, for brain and CNS cancers, inequalities in survival were greater for women (S6 Table).

Excluding cases living outside major cities did not alter the EMRRs for most cancer types, except for stomach and kidney cancer, for which inequalities in survival diminished slightly, whereas for melanoma and mesothelioma, the observed gaps in survival widened slightly (S7 Table).

## Discussion

Residents of more disadvantaged areas, relative to those residing in less disadvantaged regions, had lower survival for 21 of 29 cancer types, with inter-quintile EMRRs as high as 1.40 (95%CI 1.32–1.49), 1.91 (95%CI 1.60–2.28), 1.76 (95%CI 1.55–1.99), 1.73 (95%CI 1.46–2.04) for colorectal cancer, melanoma, female breast and prostate cancer, respectively. These inequalities persisted over the 15-year period for most cancers and widened for head and neck cancer. The gaps in survival were generally greater for younger individuals (aged <55 years) with cancers of bladder, connective/soft tissue and unknown primary.

For most cancers, socio-economic inequalities in survival generally remained unchanged after excluding cases living outside major cities, except for stomach and kidney cancer, for which the excess risk of death in more deprived areas decreased, whereas for melanoma and mesothelioma, it increased.

Strengths of this analysis include the use of SEIFA-specific life tables, the population-based design, and the large cohort of cases with complete follow up through national death registries that allowed us to examine variation in the EMRR by year of diagnosis, age at cancer diagnosis, time since diagnosis and sex. To the best of our knowledge, of the existing Australian studies of socio-economic inequalities in cancer survival, ours is the only study to use SEIFA-specific life tables. Socio-economically more disadvantaged people tend to have higher mortality due to other causes (i.e. non-cancer related deaths); [27] thus, using life tables not stratified by socio-economic disadvantage generates ‘non-comparability’ bias due to underestimation of expected mortality of more disadvantaged cancer patients and over estimation of their excess risk of death due to cancer. [25]

The main limitation was the unavailability of individual-level data on socio-economic position and the possibility of patients moving between areas with different socio-economic profiles, which could have underestimated the influence of SEP on cancer survival through misclassification. In addition, we did not have information regarding potential mediators of survival such as health-related lifestyle behaviours, stage at diagnosis, co-morbidities and cancer treatment. Thus, we were unable to investigate the potential role of these factors in explaining socio-economic inequalities in cancer survival. There is also a possibility of non-comparability bias for smoking-associated cancers as we lacked data on smoking and the life tables were not stratified by smoking status; therefore, we may have modestly overestimated socio-economic differences in survival. [25]

Our findings are consistent with those from other studies conducted in Australia, irrespective of how SEP was defined. A study using cancer registry data from New South Wales (NSW), Australia, found that with and without adjustment for stage at diagnosis, cancer patients living in more disadvantaged areas had lower survival compared with those residing in less disadvantaged areas. [28] The largest differences in survival were for melanoma and liver, prostate, lung, colorectum and breast cancer. The authors also noted that socio-economic inequalities in cancer survival had widened during the study period (1980–2008). [28] They postulated that variations in health-related behaviours, co-morbidities, and access to diagnostic and treatment facilities may explain these differences. [28] Other research conducted in NSW found persistent patterns over time of lower survival in more disadvantaged neighbourhoods, particularly for colorectal, prostate, breast, liver and stomach cancer. [10] Adjusting for a potential mediator, stage of disease, did not markedly change the results; therefore, the authors suggested quality of treatment and patient characteristics such as smoking, alcohol consumption and co-morbidities as other potential explanatory factors. [10]

Also consistent with these findings are those from studies in other Western countries. A study conducted in the United States, using the Surveillance, Epidemiology, and End Results cancer registry data, examined socio-economic inequalities in cancer survival and their temporal trends from 1950 to 2014. [29] Survival was lower for cancer patients living in more disadvantaged regions; these inequalities remained after adjusting for stage at diagnosis and widened over the six decades for colorectal, prostate and breast cancer. The authors postulated that higher prevalence of unhealthy behaviours such as smoking, excessive alcohol drinking, physical inactivity, poor diet, lower uptake of screening, lack of health insurance and limited access to healthcare and treatment services for cancer patients from lower socio-economic areas may explain the observed gaps in survival. [29] A population-based study from Canada found a similar pattern of lower cancer survival for cases from disadvantaged communities, relative to those from affluent regions, for breast, lung, colorectal and head and neck cancer. [30] The investigators observed, over 13 years, more gains in cancer survival for privileged people, which could increase inequalities in survival and proposed differences in use of screening and receiving optimal treatment as potential explanatory factors. [30] Findings from European and New Zealand research have also been consistent. A Danish study found differences between low-income and high-income patients in five-year survival for most cancers from 1987 to 2009, and that these had widened in the most recent five years, partly due to greater gains in cancer survival by affluent cases. [31] Similarly, a New Zealand study reported lower survival for low income cancer cases compared with high-income patients; the authors also found that the observed gaps in survival has widened over 13 years. [32] Another study from New Zealand observed socio-economic inequalities in cancer survival, which were only partly explained by extent of disease at diagnosis. [33] Research conducted in England and Wales [34] and Scotland [35] also found a deprivation gap in survival for several cancers comparing residents of more deprived areas with more affluent regions. Both studies observed an increase in these inequalities over 15 years. [34, 35]

Except for head and neck cancers, we observed a persistent pattern of lower survival in more disadvantaged areas over the past 15 years for most cancers, while other studies reported widening gaps over time, particularly for common cancers such as breast, prostate, colorectum and lung. [29–31, 34, 35] The causes of increasing gaps in survival are not well-understood, but it might be explained by greater improvements in cancer survival for advantaged individuals due to better access to diagnostic facilities and improved treatments, including clinical trials. [30, 36]

We found that socio-economic inequalities in survival were present across all age groups for most cancers, although the magnitude and pattern varied. A study from England examined the influence of age at diagnosis on socio-economic differences in survival from three common cancers including breast, lung and colon. [37] The authors found that the deprivation gap as measured by the absolute difference in 1-year relative survival increased with increasing age at diagnosis for breast cancer, while the opposite pattern was observed for 1- and 5-year relative survival for lung cancer. For colon cancer, it appeared that short and longer-term survival was lower for older disadvantaged patients than younger counterparts. It is not clear why socio-economic inequalities in survival varied by age at diagnosis, but these patterns may be due to differential access to diagnostic services, screening and optimal treatment across age groups. [37]

For a few cancers (pancreas, ovary, lung, brain/CNS, female breast and leukaemia), the gaps in survival decreased with time following diagnosis, which might be explained if there are variations in receiving timely treatment across socio-economic groups. It is unclear why socio-economic differences in colorectal cancer survival would be greater for men, or those for brain and CNS cancers would be greater for women.

The observed socio-economic inequalities in survival from cancers of the cervix, colorectum and female breast might be partly due to variation in participation in screening programs by socio-economic disadvantage. People living in more disadvantaged areas have lower participation in the bowel and cervical cancer screening programs than those from less disadvantaged areas. [38, 39] Variation in participation in the breast screening program is smaller across socio-economic groups, although it is slightly lower for women from the most disadvantaged areas (51.8%) than for those living in the least disadvantaged regions (55.2%). [40] While Australia has no organised screening program for prostate cancer, the prostate-specific antigen test (PSA) is widely used, especially by men of higher socio-economic position. [41] At least part of the apparently higher survival from prostate cancer in areas of less socio-economic disadvantage is due to overdiagnosis of indolent cancers by PSA testing. [42]

Several studies have attempted to identify factors that mediate socio-economic inequalities in cancer survival, but the mediating effects of the identified factors are inconsistent within and between countries, mainly due to limitations of the data and applied methods and different health care systems. Further, the applied methods to identify mediators have been suboptimal. Most studies compared relative risk estimates with and without adjusting for intermediate variables in causal pathways. This approach fails in the presence of multiple mediators that affect and interact with each other and could produce biased results. [43, 44]

The International Agency for research on cancer (IARC) recently published a comprehensive review focusing on reducing social inequalities in cancer. [5] Higher prevalence of unhealthy lifestyle behaviours, lower screening participation, advanced stage at diagnosis, and inadequate access to diagnostic and cancer treatment services among underprivileged people were reported as major contributing factors to existing inequalities in cancer outcomes. [5] Another review on the influence of socio-economic position on access to clinical trials found that cancer patients from lower socio-economic backgrounds were underrepresented in cancer treatment trials and less likely to be eligible due to multiple barriers such as presence of co-morbidities and financial concerns. [36]

## Conclusion

In summary, for most cancers, people residing in more socio-economically disadvantaged areas have lower survival relative to those living in less disadvantaged regions. Future research should focus on unravelling the influence of potential explanatory factors using innovative methods of mediation analysis, which may help to prioritise the factors that need to be changed to reduce inequalities in cancer survival.

## Supporting information

S1 Table Number of cases and deaths by area-level socio-economic disadvantage in Victoria, Australia, 2001–2015. (DOCX)

S2 Table Five-year age-standardised net survival by area-level socio-economic disadvantage in Victoria, Australia, 2001–2015. (DOCX)

S3 Table Five-year excess mortality rate ratios (EMRRs), by year of diagnosis, per quintile increase in socio-economic disadvantage (SEIFA). (DOCX)

S4 Table Five-year excess mortality rate ratios (EMRRs), by time since diagnosis, per quintile increase in socio-economic disadvantage (SEIFA). (DOCX)

S5 Table Five-year excess mortality rate ratios (EMRRs), by age at diagnosis, per quintile increase in socio-economic disadvantage (SEIFA). (DOCX)

S6 Table Five-year excess mortality rate ratios (EMRRs), by sex, per quintile increase in socio-economic disadvantage (SEIFA). (DOCX)

S7 Table Excess mortality rate ratios (EMRRs) within 5 years of diagnosis, including and excluding cases living outside major cities, per quintile increase in socio-economic disadvantage (SEIFA), 2001–2015. (DOCX)
