# Supplementary material for: Differences in cancer survival by area-level socio-economic disadvantage: A population-based study using cancer registry data
Source: PLoS One. 2020 Jan 30;15(1):e0228551. doi: 10.1371/journal.pone.0228551 (PMC6992207; doi:10.1371/journal.pone.0228551)
Supplement: S1 Table — (DOCX) [file pone.0228551.s001.docx]

**S1 Table.** Number of cases and deaths by area-level socio-economic disadvantage in Victoria, Australia, 2001-2015

| **ICD-10** | **Cancer site** | **Q1 (Least disadvantaged)** | | |  | | **Q2** | | |  | | **Q3** | | | |  | | **Q4** | | |  | | **Q5 (Most disadvantaged)** | |  |
| --- | --- | --- | --- | --- | --- | --- | --- | --- | --- | --- | --- | --- | --- | --- | --- | --- | --- | --- | --- | --- | --- | --- | --- | --- | --- |
|  |  | **Cases** | **Deaths** |  | | **Cases** | | **Deaths** |  | | **Cases** | | **Deaths** | |  | | **Cases** | | **Deaths** |  | | **Cases** | | **Deaths** | |
| C00-14, C30-32 | Head and neck | 1,719 | 552 |  | | 1,923 | | 640 |  | | 2,142 | | 802 | |  | | 2,398 | | 984 |  | | 3,034 | | 1,370 | |
| C15 | Oesophagus | 606 | 459 |  | | 675 | | 496 |  | | 717 | | 579 | |  | | 903 | | 743 |  | | 1,060 | | 866 | |
| C16 | Stomach | 1,034 | 719 |  | | 1,214 | | 857 |  | | 1,311 | | 946 | |  | | 1,471 | | 1,088 |  | | 1,798 | | 1,353 | |
| C17 | Small intestine | 227 | 92 |  | | 249 | | 122 |  | | 224 | | 103 | |  | | 223 | | 85 |  | | 282 | | 133 | |
| C18-20 | Colorectum | 7,697 | 3,243 |  | | 8,023 | | 3,493 |  | | 8,708 | | 3,928 | |  | | 9,588 | | 4,602 |  | | 10,487 | | 5,269 | |
| C21 | Anus and anal canal | 215 | 66 |  | | 190 | | 77 |  | | 203 | | | 81 |  | | 232 | | 95 |  | | 243 | | 119 | |
| C22 | Liver | 653 | 502 |  | | 783 | | 609 |  | | 805 | | | 619 |  | | 937 | | 746 |  | | 1371 | | 1,079 | |
| C23-24 | Gallbladder and biliary tract | 349 | 258 |  | | 362 | | 283 |  | | 451 | | | 352 |  | | 428 | | 339 |  | | 604 | | 481 | |
| C25 | Pancreas | 1,365 | 1,182 |  | | 1,384 | | 1,198 |  | | 1,454 | | | 1,277 |  | | 1,641 | | 1,459 |  | | 1,900 | | 1,699 | |
| C33-34 | Lung, bronchus and trachea | 3,851 | 3,127 |  | | 4,518 | | 3,646 |  | | 5,335 | | | 4,395 |  | | 6,577 | | 5,493 |  | | 8,701 | | 7,307 | |
| C43 | Melanoma | 6,800 | 1,130 |  | | 5,862 | | 1,128 |  | | 5,802 | | | 1,220 |  | | 5,553 | | 1,346 |  | | 4,650 | | 1,268 | |
| C45 | Mesothelioma | 299 | 262 |  | | 301 | | 270 |  | | 365 | | | 329 |  | | 359 | | 328 |  | | 392 | | 362 | |
| C47-49 | Connective and soft tissue | 411 | 128 |  | | 424 | | 174 |  | | 375 | | | 145 |  | | 378 | | 171 |  | | 365 | | 181 | |
| C50 | Female breast | 10,510 | 1,671 |  | | 9,612 | | 1,666 |  | | 9,326 | | | 1,867 |  | | 9,104 | | 2,018 |  | | 8,991 | | 2,176 | |
| C53 | Cervix | 394 | 87 |  | | 439 | | 109 |  | | 444 | | | 124 |  | | 505 | | 154 |  | | 587 | | 184 | |
| C54-55 | Uterus | 1,311 | 278 |  | | 1,373 | | 293 |  | | 1,416 | | | 307 |  | | 1,480 | | 333 |  | | 1,708 | | 433 | |
| C56 | Ovary | 816 | 475 |  | | 813 | | 463 |  | | 866 | | | 521 |  | | 896 | | 577 |  | | 916 | | 581 | |
| C51-52, C57 | Vulva, vagina, other/ unspecified | 267 | 120 |  | | 259 | | 99 |  | | 299 | | | 121 |  | | 314 | | 147 |  | | 389 | | 157 | |
| C61 | Prostate | 12,722 | 2,366 |  | | 11,519 | | 2,596 |  | | 11,150 | | | 2,787 |  | | 11,059 | | 3,221 |  | | 10,797 | | 3,439 | |
| C64 | Kidney | 1,403 | 454 |  | | 1,492 | | 494 |  | | 1,572 | | | 519 |  | | 1,783 | | 634 |  | | 1,996 | | 849 | |
| C67 | Bladder | 1,075 | 582 |  | | 1,146 | | 609 |  | | 1,277 | | | 701 |  | | 1,463 | | 838 |  | | 1,722 | | 1,014 | |
| C65-66, C68 | Renal pelvis, ureter, other/unspecified | 167 | 94 |  | | 207 | | 113 |  | | 205 | | | 120 |  | | 258 | | 176 |  | | 292 | | 184 | |
| C70-72 | Brain and central nervous system | 1,053 | 731 |  | | 1,073 | | 786 |  | | 1,070 | | | 772 |  | | 1,046 | | 815 |  | | 1,034 | | 783 | |
| C73 | Thyroid | 1,154 | 65 |  | | 1,119 | | 100 |  | | 1,058 | | | 100 |  | | 1,003 | | 121 |  | | 1,058 | | 116 | |
| C80 | Unknown primary | 959 | 806 |  | | 1,131 | | 970 |  | | 1,337 | | | 1,169 |  | | 1,584 | | 1,414 |  | | 1,994 | | 1,780 | |
| C81 | Hodgkin Lymphoma | 421 | 47 |  | | 407 | | 53 |  | | 424 | | | 56 |  | | 387 | | 68 |  | | 402 | | 84 | |
| C82-86 | Non-Hodgkin Lymphoma | 3,012 | 966 |  | | 2,771 | | 905 |  | | 2,805 | | | 1,058 |  | | 2,881 | | 1,108 |  | | 3,026 | | 1,276 | |
| C90 | Multiple Myeloma | 972 | 506 |  | | 925 | | 500 |  | | 976 | | | 552 |  | | 1,041 | | 615 |  | | 1,107 | | 690 | |
| C91-95 | Leukemia | 1,595 | 719 |  | | 1,636 | | 804 |  | | 1,680 | | | 848 |  | | 1,789 | | 985 |  | | 1,920 | | 1,071 | |
