# Supplementary material for: Differences in cancer survival by area-level socio-economic disadvantage: A population-based study using cancer registry data
Source: PLoS One. 2020 Jan 30;15(1):e0228551. doi: 10.1371/journal.pone.0228551 (PMC6992207; doi:10.1371/journal.pone.0228551)
Supplement: S2 Table — (DOCX) [file pone.0228551.s002.docx]

**S2 Table.** Five-year age-standardised net survival by area-level socio-economic disadvantage in Victoria, Australia, 2001-2015

|  | | Q1 (Least disadvantaged) | Q2 | Q3 | Q4 | Q5 (Most Disadvantaged) |
| --- | --- | --- | --- | --- | --- | --- |
| ICD-10 | **Cancer site** | **Net survival % (95% CI)** | **Net survival % (95% CI)** | **Net survival % (95% CI)** | **Net survival % (95% CI)** | **Net survival % (95% CI)** |
| C00-C14, C30-C32 | Head and Neck | 77.6 (74.7 – 80.2) | 78.0 (75.1 – 80.6) | 74.5 (71.8 – 77.0) | 70.6 (68.0 – 73.0) | 65.9 (63.6 – 68.1) |
| C15 | Oesophagus | 25.1 (21.2 – 29.3) | 27.9 (23.9 – 32.0) | 18.8 (15.5 – 22.3) | 17.0 (14.1 – 20.1) | 17.4 (14.7 – 20.2) |
| C16 | Stomach | 32.7 (29.1 – 36.3) | 31.1 (28.0 – 34.2) | 28.0 (25.0 – 31.0) | 26.0 (23.3 – 28.7) | 27.2 (24.8 – 29.8) |
| C17 | Small intestine | 64.5 (55.7 – 72.0) | 56.8 (49.1 – 63.8) | 59.8 (51.4 – 67.2) | 66.7 (57.6 – 74.3) | 63.7 (56.1 – 70.3) |
| C18-20 | Colorectum | 71.5 (70.0 – 72.9) | 68.3 (66.8 – 69.7) | 67.0 (65.6 – 68.3) | 65.5 (64.1 – 66.8) | 62.9 (61.7 – 64.2) |
| C21 | Anal and anal canal | 74.5 (65.5 – 81.5) | 71.1 (61.2 – 79.0) | 67.9 (59.4 – 75.1) | 69.3 (60.1 – 76.8) | 61.5 (53.5 – 68.5) |
| C22 | Liver | 19.7 (16.3 – 23.3) | 18.6 (15.4 – 21.9) | 16.8 (13.8 – 20.0) | 15.4 (12.6 – 18.5) | 16.8 (14.5 – 19.4) |
| C23-C24 | Gallbladder and biliary tract | 22.5 (17.4 – 28.0) | NA | 24.8 (20.2 – 29.7) | 18.5 (14.3 – 23.2) | 19.8 (16.0 – 23.8) |
| C25 | Pancreas | 8.7 (7.0 – 10.6) | 9.6 (7.7 – 11.7) | 7.7 (6.1 – 9.5) | 6.5 (5.2 – 8.1) | 8.7 (7.2 – 10.4) |
| C33-C34 | Lung, bronchus, and trachea | 17.7 (16.3 – 19.2) | 17.4 (16.1 – 18.7) | 16.8 (15.6 – 18.0) | 15.3 (14.2 – 16.4) | 14.2 (13.3 – 15.1) |
| C43 | Melanoma | 93.2 (91.8 – 94.4) | 92.0 (90.6 – 93.3) | 90.6 (89.1 – 91.9) | 89.3 (87.8 – 90.6) | 88.4 (86.7 – 89.9) |
| C45 | Mesothelioma | 7.9 (4.8 – 12.1) | 7.5 (4.4 – 11.5) | NA | 4.8 (2.7 – 7.8) | NA |
| C47-C49 | Connective and soft tissue | 74.2 (67.8 – 79.5) | 65.9 (59.5 – 71.5) | 67.4 (60.4 – 73.5) | 63.4 (57.0 – 69.2) | 58.6 (51.8 – 64.8) |
| C50 | Female Breast | 92.6 (91.6 – 93.5) | 90.4 (89.4 – 91.4) | 89.6 (88.6 – 90.6) | 89.1 (88.0 – 90.1) | 87.7 (86.7 – 88.7) |
| C53 | Cervix | 77.2 (72.4 – 81.3) | 76.1 (71.4 – 80.2) | 74.9 (70.0 – 79.1) | 72.4 (67.8 – 76.5) | 74.3 (70.2 – 78.0) |
| C54-C55 | Uterus | 87.1 (84.1 – 89.5) | 86.2 (83.4 – 88.6) | 85.4 (82.4 – 87.9) | 85.2 (82.4 – 87.6) | 84.0 (81.5 – 86.3) |
| C56 | Ovary | 44.1 (40.4 – 47.7) | 42.5 (38.8 – 46.1) | 41.7 (38.0 – 45.3) | 38.9 (35.3 – 42.1) | 40.7 (37.2 – 44.2) |
| C51-C52- C57 | Vulva, vagina, other/unspecified | 61.6 (53.3 – 68.9) | 73.9 (64.6 – 81.1) | 65.8 (578.4 – 72.9) | 60.2 (53.0 – 66.7) | 63.9 (56.8 – 70.2) |
| C61 | Prostate | 94.1 (93.1 – 94.9) | 93.3 (92.3 – 94.2) | 92.3 (91.2 – 93.2) | 91.4 (90.3 – 92.3) | 90.2 (89.1 – 91.2) |
| C64 | Kidney | 74.7 (71.6 – 77.5) | 72.9 (69.9 – 75.7) | 74.7 (71.6 – 77.4) | 73.6 (70.9 – 76.1) | 68.0 (65.4 – 70.4) |
| C67 | Bladder | 57.8 (53.7 – 61.8) | 54.7 (50.5 – 58.8) | 54.0 (50.0 – 57.8) | 52.1 (48.2 – 55.8) | 49.0 (45.5 – 52.4) |
| C65-C66-C68 | Renal pelvis, ureter, other/unspecified urinary organs | 51.1 (41.6 – 59.8) | 44.5 (35.5 – 53.1) | 39.8 (32.1 – 47.4) | 39.8 (32.1 – 47.4) | 37.8 (30.7 – 44.9) |
| C70-C72 | Brain and CNS | 26.0 (23.5 – 28.6) | 24.0 (21.5 – 26.5) | 24.6 (22.1 – 27.1) | 22.4 (19.9 – 25.0) | 23.7 (21.2 – 26.3) |
| C73 | Thyroid | 96.6 (94.1 – 98.0) | 94.9 (92.6 – 96.4) | 94.8 (92.2 – 96.5) | 94.0 (91.5 – 95.7) | 93.9 (91.3 – 95.7) |
| C80 | Unknown primary | 16.9 (14.3 – 19.6) | 16.8 (14.4 – 19.4) | 13.3 (11.2 – 15.5) | 13.4 (11.4 – 15.5) | 12.6 (10.9 – 14.4) |
| C81 | Hodgkin Lymphoma | 89.7 (85.8 – 92.6) | 89.4 (85.9 – 92.0) | 88.5 (84.4 – 91.6) | 88.6 (85.0 – 91.5) | 86.2 (82.5 – 89.1) |
| C82-85 | Non-Hodgkin Lymphoma | 77.8 (75.6 – 79.8) | 75.8 (73.5 – 78.0) | 72.7 (70.4 – 74.7) | 71.5 (69.3 – 73.7) | 69.0 (66.8 – 71.1) |
| C90 | Multiple Myeloma | 50.8 (46.5 – 55.0) | 46.8 (42.8 – 50.7) | 50.0 (45.9 – 53.9) | 45.6 (41.8 – 49.4) | 47.3 (43.4 – 51.2) |
| C91-C95 | Leukaemia | 53.8 (50.6 – 56.8) | 52.7 (49.6 – 55.6) | 54.1 (51.0 – 57.0) | 53.0 (50.1 – 55.8) | 52.4 (49.6 – 55.1) |

CI, confidence interval; NA, not available (age-standardised net survival cannot be estimated)
