# Supplementary material for: Differences in cancer survival by area-level socio-economic disadvantage: A population-based study using cancer registry data
Source: PLoS One. 2020 Jan 30;15(1):e0228551. doi: 10.1371/journal.pone.0228551 (PMC6992207; doi:10.1371/journal.pone.0228551)
Supplement: S3 Table — (DOCX) [file pone.0228551.s003.docx]

**S3 Table.** Five-year excess mortality rate ratios (EMRRs), by year of diagnosis, per quintile increase in socio-economic disadvantage (SEIFA)

|  | Year of diagnosis | | | | | | |
| --- | --- | --- | --- | --- | --- | --- | --- |
|  |  | **2001-2005** | **2006-2010** | **2011-2015** | **P-trend^** | **P-departure from linearity^** |  |
| ICD-10 | **Cancer site** | **EMRR (95% CI)** | **EMRR (95% CI)** | **EMRR (95% CI)** |  |  |  |
| C00-14, C30-32 | Head and neck | 1.10 (1.04, 1.15) | 1.17 (1.11, 1.23) | 1.19 (1.12, 1.26) | 0.03 | 0.3 |  |
| C15 | Oesophagus | 1.09 (1.04, 1.14) | 1.10 (1.05, 1.15) | 1.02 (0.97, 1.07) | 0.06 | 0.3 |  |
| C16 | Stomach | 1.05 (1.01, 1.09) | 1.07 (1.03, 1.11) | 1.02 (0.98, 1.06) | 0.4 | 0.3 |  |
| C18-20 | Colorectum | 1.07 (1.05, 1.09) | 1.10 (1.07, 1.12) | 1.07 (1.04, 1.10) | 0.6 | 0.003 |  |
| C21 | Anus and anal canal | 1.23 (1.05, 1.45) | 1.15 (1.00, 1.33) | 1.00 (0.86, 1.17) | 0.1 | 0.9 |  |
| C22 | Liver | 1.03 (0.99, 1.08) | 1.03 (0.99, 1.07) | 1.04 (1.00, 1.09) | 0.8 | 0.9 |  |
| C23-24 | Gallbladder and biliary tract | 1.06 (1.00, 1.13) | 1.04 (0.98, 1.10) | 1.04 (0.97, 1.11) | 0.7 | 1.0 |  |
| C25 | Pancreas | 1.04 (1.01, 1.08) | 1.04 (1.01, 1.07) | 1.08 (1.05, 1.11) | 0.1 | 0.03 |  |
| C33-34 | Lung, bronchus, and trachea | 1.04 (1.02, 1.06) | 1.04 (1.03, 1.06) | 1.05 (1.03, 1.07) | 0.3 | 0.4 |  |
| C43 | Melanoma | 1.16 (1.09, 1.25) | 1.20 (1.13, 1.27) | 1.19 (1.10, 1.29) | 0.6 | 0.2 |  |
| C47-49 | Connective and soft tissue | 1.08 (0.97, 1.21) | 1.10 (0.99, 1.22) | 1.10 (0.98, 1.23) | 0.9 | 0.7 |  |
| C50 | Female breast | 1.13 (1.08, 1.18) | 1.12 (1.07, 1.17) | 1.20 (1.12, 1.27) | 0.3 | 0.6 |  |
| C56 | Ovary | 1.10 (1.05, 1.15) | 1.05 (1.00, 1.10) | 1.04 (0.98, 1.11) | 0.2 | 0.08 |  |
| C61 | Prostate | 1.13 (1.07, 1.18) | 1.19 (1.11, 1.27) | 1.09 (0.98, 1.20) | 0.1 | 0.001 |  |
| C64 | Kidney | 1.07 (1.01, 1.13) | 1.07 (1.01, 1.13) | 1.05 (0.98, 1.12) | 0.7 | 1.0 |  |
| C67 | Bladder | 1.07 (1.02, 1.13) | 1.08 (1.03, 1.14) | 1.05 (0.99, 1.12) | 0.7 | 0.6 |  |
| C70-72 | Brain and central nervous system | 1.03 (0.99, 1.07) | 1.05 (1.02, 1.10) | 1.09 (1.04, 1.14) | 0.08 | 0.7 |  |
| C80 | Unknown primary | 1.06 (1.02, 1.09) | 1.09 (1.05, 1.12) | 1.09 (1.05, 1.13) | 0.1 | 0.05 |  |
| C82-86 | Non-Hodgkin lymphoma | 1.13 (1.08, 1.18) | 1.12 (1.07, 1.17) | 1.05 (1.00, 1.11) | 0.07 | 0.1 |  |
| C90 | Multiple myeloma | 1.01 (0.96, 1.07) | 1.05 (1.00, 1.11) | 1.08 (1.01, 1.15) | 0.2 | 0.1 |  |
| C91-95 | Leukaemia | 1.05 (1.00, 1.09) | 1.02 (0.98, 1.06) | 1.03 (0.98, 1.08) | 0.7 | 0.7 |  |

CI, confidence interval; ^ likelihood ratio test; SEIFA, Socio-Economic Indexes for Areas
