# Supplementary material for: Differences in cancer survival by area-level socio-economic disadvantage: A population-based study using cancer registry data
Source: PLoS One. 2020 Jan 30;15(1):e0228551. doi: 10.1371/journal.pone.0228551 (PMC6992207; doi:10.1371/journal.pone.0228551)
Supplement: S5 Table — (DOCX) [file pone.0228551.s005.docx]

**S5 Table.** Five-year excess mortality rate ratios (EMRRs), by age at diagnosis, per quintile increase in socio-economic disadvantage (SEIFA)

|  | |  |  | Age at diagnosis (years) | | | | | | | | |
| --- | --- | --- | --- | --- | --- | --- | --- | --- | --- | --- | --- | --- |
|  | |  | | | **15-44** | **45-54** | **55-64** | **65-74** | **75+** | **P-trend^** | **P-departure from linearity^** |  |
| ICD-10 | | **Cancer site** | | | **EMRR (95% CI)** | **EMRR (95% CI)** | **EMRR (95% CI)** | **EMRR (95% CI)** | **EMRR (95% CI)** |  |  |  |
| C00-14, C30-32 | Head and neck | | | | 1.19 (1.03, 1.37) | 1.19 (1.09, 1.29) | 1.19 (1.12, 1.26) | 1.13 (1.06, 1.20) | 1.09 (1.03, 1.16) | 0.05 | 0.9 |  |
| C15 | Oesophagus | | | | 1.05 (0.88, 1.26) | 1.12 (1.02, 1.22) | 1.09 (1.03, 1.16) | 1.09 (1.04, 1.15) | 1.05 (1.01, 1.09) | 0.08 | 0.9 |  |
| C16 | Stomach | | | | 1.06 (0.96, 1.16) | 1.05 (0.98, 1.13) | 1.01 (0.96, 1.07) | 1.05 (1.00, 1.09) | 1.06 (1.02, 1.10) | 0.4 | 0.7 |  |
| C18-20 | Colorectum | | | | 1.12 (1.05, 1.19) | 1.09 (1.05, 1.14) | 1.08 (1.04, 1.11) | 1.09 (1.06, 1.12) | 1.07 (1.05, 1.10) | 0.2 | 0.9 |  |
| C21 | Anus and anal canal | | | | 1.75 (1.13, 2.72) | 1.02 (0.84, 1.23) | 1.13 (0.94, 1.35) | 1.24 (1.01, 1.51) | 1.04 (0.88, 1.22) | 0.4 | 0.1 |  |
| C22 | Liver | | | | 1.08 (0.94, 1.24) | 1.03 (0.96, 1.10) | 1.08 (1.03, 1.14) | 0.99 (0.95, 1.04) | 1.04 (1.00, 1.08) | 0.3 | 0.2 |  |
| C23-24 | Gallbladder and biliary tract | | | | 0.91 (0.71, 1.18) | 1.09 (0.95, 1.24) | 1.02 (0.94, 1.11) | 1.04 (0.97, 1.11) | 1.07 (1.01, 1.13) | 0.4 | 0.7 |  |
| C25 | Pancreas | | | | 1.02 (0.91, 1.14) | 1.06 (0.99, 1.12) | 1.05 (1.01, 1.09) | 1.06 (1.03, 1.09) | 1.05 (1.03, 1.08) | 0.7 | 1.0 |  |
| C33-34 | Lung, bronchus, and trachea | | | | 1.13 (1.04, 1.21) | 1.05 (1.01, 1.08) | 1.05 (1.02, 1.07) | 1.05 (1.03, 1.07) | 1.03 (1.02, 1.05) | 0.05 | 0.3 |  |
| C43 | Melanoma | | | | 1.26 (1.14, 1.39) | 1.17 (1.07, 1.29) | 1.17 (1.08, 1.27) | 1.23 (1.12, 1.35) | 1.12 (1.04, 1.21) | 0.2 | 0.5 |  |
| C47-49 | Connective and soft tissue | | | | 1.23 (1.06, 1.41) | 1.27 (1.05, 1.54) | 1.05 (0.90, 1.24) | 1.01 (0.89, 1.16) | 1.04 (0.93, 1.17) | 0.03 | 0.6 |  |
| C50 | Female breast | | | | 1.06 (1.00, 1.13) | 1.15 (1.09, 1.22) | 1.17 (1.10, 1.24) | 1.18 (1.09, 1.26) | 1.12 (1.05, 1.19) | 0.3 | 0.1 |  |
| C56 | Ovary | | | | 1.12 (0.99, 1.27) | 1.05 (0.96, 1.15) | 1.05 (0.98, 1.12) | 1.07 (1.01, 1.13) | 1.08 (1.02, 1.13) | 0.8 | 0.8 |  |
| C61 | Prostate | | | | 1.00 (0.57, 1.77) | 1.46 (1.18, 1.80) | 1.20 (1.07, 1.36) | 1.37 (1.21, 1.54) | 1.09 (1.05, 1.14) | <0.001 | 0.01 |  |
| C64 | Kidney | | | | 1.01 (0.87, 1.17) | 1.09 (0.99, 1.20) | 1.12 (1.04, 1.20) | 1.07 (0.99, 1.15) | 1.04 (0.98, 1.10) | 0.5 | 0.5 |  |
| C67 | Bladder | | | | 1.19 (0.90, 1.58) | 1.22 (1.05, 1.40) | 1.15 (1.06, 1.25) | 1.08 (1.02, 1.15) | 1.04 (1.00, 1.08) | 0.004 | 0.9 |  |
| C70-72 | Brain and central nervous system | | | | 1.02 (0.95, 1.10) | 1.07 (1.01, 1.14) | 1.00 (0.96, 1.05) | 1.10 (1.05, 1.15) | 1.07 (1.02, 1.12) | 0.2 | 0.1 |  |
| C80 | Unknown primary | | | | 1.14 (1.00, 1.29) | 1.14 (1.06, 1.23) | 1.10 (1.04, 1.16) | 1.11 (1.07, 1.15) | 1.04 (1.02, 1.07) | 0.005 | 0.6 |  |
| C82-86 | Non-Hodgkin lymphoma | | | | 1.08 (0.97, 1.20) | 1.07 (0.98, 1.18) | 1.12 (1.05, 1.20) | 1.16 (1.10, 1.22) | 1.08 (1.04, 1.12) | 0.9 | 0.3 |  |
| C90 | Multiple myeloma | | | | 0.97 (0.75, 1.27) | 1.09 (0.96, 1.25) | 1.08 (1.00, 1.17) | 1.01 (0.95, 1.07) | 1.05 (1.00, 1.10) | 0.7 | 0.6 |  |
| C91-95 | Leukaemia | | | | 1.08 (0.98, 1.18) | 1.08 (0.99, 1.19) | 1.00 (0.94, 1.07) | 1.06 (1.01, 1.11) | 1.01 (0.98, 1.05) | 0.2 | 0.4 |  |

CI, confidence interval; ^ likelihood ratio test; SEIFA, Socio-Economic Indexes for Areas
