# Supplementary material for: Differences in cancer survival by area-level socio-economic disadvantage: A population-based study using cancer registry data
Source: PLoS One. 2020 Jan 30;15(1):e0228551. doi: 10.1371/journal.pone.0228551 (PMC6992207; doi:10.1371/journal.pone.0228551)
Supplement: S7 Table — (DOCX) [file pone.0228551.s007.docx]

**S7 Table.** Excess mortality rate ratios (EMRRs) within 5 years of diagnosis, including and excluding cases living outside major cities, per quintile increase in socio-economic disadvantage (SEIFA), 2001-2015

|  |  |  |  |  |  | |
| --- | --- | --- | --- | --- | --- | --- |
| **ICD-10** | **Cancer site** | **EMRR (95% CI) ^$^** | **P-value ^** | **EMRR (95% CI) *** | **P-value ^** |  |
| C00-14, C30-32 | Head and neck | 1.15 (1.11 to 1.18) | <0.001 | 1.13 (1.08 to 1.17) | <0.001 |  |
| C15 | Oesophagus | 1.07 (1.04 to 1.10) | <0.001 | 1.06 (1.03 to 1.10) | 0.001 |  |
| C16 | Stomach | 1.05 (1.02 to 1.07) | <0.001 | 1.02 (1.00 to 1.05) | 0.09 |  |
| C17 | Small intestine | 1.00 (0.93 to 1.07) | 1.0 | 1.00 (0.92 to 1.09) | 1.0 |  |
| C18-20 | Colorectum | 1.08 (1.07 to 1.10) | <0.001 | 1.08 (1.06 to 1.10) | <0.001 |  |
| C21 | Anus and anal canal | 1.13 (1.04 to 1.23) | 0.006 | 1.15 (1.03 to 1.27) | 0.01 |  |
| C22 | Liver | 1.04 (1.01 to 1.06) | 0.004 | 1.03 (1.00 to 1.06) | 0.07 |  |
| C23-24 | Gallbladder and biliary tract | 1.05 (1.01 to 1.09) | 0.008 | 1.05 (1.01 to 1.09) | 0.02 |  |
| C25 | Pancreas | 1.05 (1.04 to 1.07) | <0.001 | 1.04 (1.02 to 1.06) | <0.001 |  |
| C33-34 | Lung, bronchus and trachea | 1.04 (1.03 to 1.05) | <0.001 | 1.04 (1.03 to 1.05) | <0.001 |  |
| C43 | Melanoma | 1.18 (1.14 to 1.23) | <0.001 | 1.22 (1.16 to 1.28) | <0.001 |  |
| C45 | Mesothelioma | 1.03 (1.00 to 1.07) | 0.07 | 1.06 (1.01 to 1.11) | 0.02 |  |
| C47-49 | Connective and soft tissue | 1.10 (1.03 to 1.17) | 0.004 | 1.07 (0.99 to 1.16) | 0.09 |  |
| C50 | Female breast | 1.14 (1.10 to 1.17) | <0.001 | 1.15 (1.12 to 1.19) | <0.001 |  |
| C53 | Cervix | 1.03 (0.97 to 1.10) | 0.4 | 1.04 (0.97 to 1.12) | 0.3 |  |
| C54-55 | Uterus | 1.04 (0.99 to 1.10) | 0.1 | 1.02 (0.96 to 1.09) | 0.5 |  |
| C56 | Ovary | 1.07 (1.04 to 1.10) | <0.001 | 1.05 (1.01 to 1.09) | 0.009 |  |
| C51-52, C57 | Vulva, vagina, other/unspecified | 1.03 (0.96 to 1.11) | 0.4 | 1.06 (0.97 to 1.16) | 0.2 |  |
| C61 | Prostate | 1.14 (1.10 to 1.18) | <0.001 | 1.10 (1.05 to 1.15) | <0.001 |  |
| C64 | Kidney | 1.07 (1.03 to 1.10) | <0.001 | 1.03 (0.99 to 1.07) | 0.2 |  |
| C67 | Bladder | 1.07 (1.04 to 1.10) | <0.001 | 1.07 (1.03 to 1.10) | 0.001 |  |
| C65-66, C68 | Renal pelvis, ureter, other/unspecified | 1.07 (1.00 to 1.14) | 0.05 | 1.08 (1.00 to 1.17) | 0.05 |  |
| C70-72 | Brain and central nervous system | 1.05 (1.03 to 1.08) | <0.001 | 1.03 (1.00 to 1.06) | 0.03 |  |
| C73 | Thyroid | 1.06 (0.95 to 1.17) | 0.3 | 1.05 (0.92 to 1.18) | 0.5 |  |
| C80 | Unknown primary | 1.07 (1.05 to 1.09) | <0.001 | 1.09 (1.06 to 1.11) | <0.001 |  |
| C81 | Hodgkin Lymphoma | 1.07 (0.96 to 1.19) | 0.2 | 1.07 (0.94 to 1.21) | 0.3 |  |
| C82-86 | Non-Hodgkin Lymphoma | 1.10 (1.07 to 1.13) | <0.001 | 1.10 (1.07 to 1.14) | <0.001 |  |
| C90 | Multiple Myeloma | 1.04 (1.01 to 1.08) | 0.01 | 1.03 (0.99 to 1.07) | 0.1 |  |
| C91-95 | Leukemia | 1.03 (1.01 to 1.07) | 0.01 | 1.04 (1.01 to 1.07) | 0.02 |  |

CI, confidence interval; ^ likelihood ratio test; ^$^ EMRRS were estimated including all cases; *EMRRs were estimated excluding cases living outside major cities
